# Formulation of Isopropyl Isothiocyanate Loaded Nano Vesicles Delivery Systems: In Vitro Characterization and In Vivo Assessment

**DOI:** 10.3390/molecules27092876

**Published:** 2022-04-30

**Authors:** Chandra Kala, Mohammad Asif, Sadaf Jamal Gilani, Syed Sarim Imam, Najam Ali Khan, Mohamad Taleuzzaman, Ameeduzzafar Zafar, Mohammed Muqtader Ahmed, Sultan Alshehri, Mohammed M. Ghoneim

**Affiliations:** 1 Department of Pharmacology, Faculty of Pharmacy, Maulana Azad University, Jodhpur 342802, Rajasthan, India; 2 Faculty of Pharmacy, Lachoo Memorial College of Science and Technology, Sector-A, Shastri Nagar, Jodhpur 342001, Rajasthan, India; writemail2asif@gmail.com; 3 Department of Basic Health Sciences, Preparatory Year, Princess Nourah Bint Adbulrahman University, Riyadh 11671, Saudi Arabia; sjglani@pnu.edu.sa; 4 Department of Pharmaceutics, College of Pharmacy, King Saud University, Riyadh 11451, Saudi Arabia; salshehri1@ksu.edu.sa; 5 GMS College of Pharmacy, Shakarpur, Rajabpur, Amroha 244236, Uttar Pradesh, India; alikhan_najam@yahoo.co.in; 6 Department of Pharmaceutical Chemistry, Faculty of Pharmacy, Maulana Azad University, Jodhpur 342802, Rajasthan, India; zzaman007@gmail.com; 7 Department of Pharmaceutics, College of Pharmacy, Jouf University, Sakaka 72341, Al-Jouf, Saudi Arabia; zzafarpharmacian@gmail.com; 8 Department of Pharmaceutics, College of Pharmacy, Prince Sattam Bin Abdulaziz University, Al-Kharj 11942, Saudi Arabia; mo.ahmed@psau.edu.sa; 9 Department of Pharmacy Practice, College of Pharmacy, Almaarefa University, Ad Diriyah 13713, Saudi Arabia; mghoneim@mcst.edu.sa

**Keywords:** isopropyl isothiocyanate, chitosan, vesicles, anti-platelet and anti-thrombotic activity

## Abstract

Isopropyl Isothiocyanate (IPI) is a poorly water-soluble drug used in different biological activities. So, the present work was designed to prepare and evaluate IPI loaded vesicles and evaluated for vesicle size, polydispersity index (PDI) and zeta potential, encapsulation efficiency, drug release, and drug permeation. The selected formulation was coated with chitosan and further assessed for the anti-platelet and anti-thrombotic activity. The prepared IPI vesicles (F3) exhibited a vesicle size of 298 nm ± 5.1, the zeta potential of −18.7 mV, encapsulation efficiency of 86.2 ± 5.3% and PDI of 0.33. The chitosan-coated IPI vesicles (F3C) exhibited an increased size of 379 ± 4.5 nm, a positive zeta potential of 23.5 ± 2.8 mV and encapsulation efficiency of 77.3 ± 4.1%. IPI chitosan vesicle (F3C) showed enhanced mucoadhesive property (2.7 folds) and intestinal permeation (~1.8-fold) higher than IPI vesicles (F3). There was a significant (*p* < 0.05) enhancement in size, muco-adhesion, and permeation flux achieved after coating with chitosan. The IPI chitosan vesicle (F3C) demonstrated an enhanced bleeding time of 525.33 ± 12.43 s, anti-thrombin activity of 59.72 ± 4.21, and inhibition of platelet aggregation 68.64 ± 3.99%, and anti-platelet activity of 99.47%. The results of the study suggest that IPI chitosan vesicles showed promising in vitro results, as well as improved anti-platelet and anti-thrombotic activity compared to pure IPI and IPI vesicles.

## 1. Introduction

The platelets play an important role in hemostasis and thrombosis. The first step in hemostasis is the activation of subendothelial matrix protein and/or endothelial cells, as well as activation of platelet at that site. This mechanism leads to an increased concentration of intracellular Ca2+ and activation of protein kinase. Various chemicals, such as thromboxane growth factors, serotonin, etc., are secreted due to platelet activation, facilitating platelet aggregation and halting blood loss. However, unwanted platelet activation, due to certain pathological conditions, may lead to enhanced platelet aggregation, resulting in thrombosis and thrombotic disorders [1].

The number of phytoconstituents has been reported to exhibit immense therapeutic potential. Isothiocyanates (ITCs), a naturally occurring compound present in cruciferous vegetables, constitute a defence system of the plant. It is a hydrolytic product of glucosinolates in the presence of the enzyme myrosinase. These are known to exhibit potent biological effects viz neuroprotection, cardioprotection, anti-inflammatory, and anti-cancer [2]. Sulforaphane and Allyl isothiocyanate has been reported to exhibit potent anti-thrombotic and anti-platelet activity [3,4]. Isopropyl isothiocyanate (IPI) is a component obtained from seed kernel and leaf of Moringa peregrina. It is a natural component of mustard seed oil. There are different pharmacological activities reported and, recently, IPI demonstrated a potent anti-platelet and anti-thrombotic activity at a dose of 30 and 40 mg/kg [5].

The encapsulation of poorly soluble drugs into lipid vesicles is an important formulation approach to increase the solubility, dissolution, bioavailability, and therapeutic efficacy. The application of lipid vesicles is a widely accepted technique for hydrophilic (propranolol Hcl, and salbutamol sulphate) [6,7], hydrophobic drugs (luteolin, risperidone, ursolic acid) [8,9,10,11]. It is composed of cholesterol, surfactant, and phospholipid, and is similar to the cell membrane [6]. It can offer prolonged drug release, increased permeation, enhanced bioavailability, and prevents drug metabolism [12,13]. 

Recently, the lipid vesicle surface was further modified to improve its stability and functionalization. The application of chitosan coating is the most common approach to enhance muco-adhesion and permeation. It also helps to increase the stability in biological fluids, as well as cellular uptake [14,15,16]. It is a deacylated linear copolymer that is obtained from chitin: a natural polysaccharide, present in the shells of crustaceans and insects [17,18]. It has many advantages, such as being non-toxic, biocompatible, and biodegradable. It also exhibits various interesting biological properties, such as facilitating the opening of the tight epithelial junction, increased circulation time, modulation of drug release, as well as drug targeting [19]. It can enhance drug absorption by improving the paracellular pathway in the intestinal tract. Various chitosan-coated lipid vesicles have been published to increase the paracellular pathway for drugs, such as thymoquinone [20], DNA vaccine [21], dopamine [22], and so on. 

To date, there is literature reporting the use of isopropyl isothiocyanate (IPI) in the delivery system. Their biological activity is reported for the pure IPI, as well as IPI extract. Therefore, the present study was designed to prepare IPI a water-insoluble molecule loaded nano-lipid vesicles. The formulations were prepared using different non-ionic surfactants with cholesterol and phospholipid. The formulations were evaluated for vesicle size, zeta potential, PDI, and encapsulation efficiency. The optimized formulation was further coated with chitosan and assessed for mucoadhesive study, permeation study, and release study. Finally, the optimized formulation (IPI chitosan vesicles) was evaluated for anti-platelet and anti-thrombotic activities.

## 2. Materials and Methods

### 2.1. Materials

Isopropyl isothiocyanate, was purchased from Sigma Aldrich, St. Louis, MO, USA. Span 20, span 40, span 60, and span 80 were purchased from SD fine chemicals, Mumbai, India. Phospholipid 90G was received as gift sample from Lipoid GmbH, Ludwigshafen, Germany. Acetylsalicylic acid (Aspirin) was purchased from CDH Fine chemicals, New Delhi, India. The other materials, thrombin, carageenan, and chromogenic substrate, were procured from Sigma Aldrich, St. Louis, MO, USA. Cholesterol was purchased from Alpha Chemika, (Mumbai, India). Methanol and chloroform was purchased from Fisher Scientific, Loughborough, UK.

### 2.2. Methods

#### 2.2.1. Formulation of Isopropyl Isothiocyanate (IPI) Vesicles

IPI vesicles were prepared by the solvent evaporation hydration method with slight modification [23]. In the first step, cholesterol, surfactants (span 20, span 40, span 60, span 80), and phospholipid were taken in the specified quantity and dissolved in methanol: chloroform (1:2, 6 mL), as shown in Table 1. Separately, IPI (50 mg) was dissolved in methanol (4 mL) followed by sonication to obtain a clear solution. Both the organic solvents were mixed in a round bottom flask and evaporated using rotary evaporator at 50 °C under reduced pressure. A dry thin film was formed on the wall of the flask after the complete removal of organic solvent. The round bottom flask was kept overnight in the desiccators to remove the residue of organic solvent. The round bottom flask was kept overnight in the desiccators for removal of organic solvent residue. The prepared thin lipid film was hydrated with 10 mL of phosphate buffer (pH 7) for 1 h. For stabilization, the vesicles were placed in the refrigerator at 4 ± 2 °C overnight.

#### 2.2.2. Formulation of Chitosan Coated Isopropyl Isothiocyanate (IPI) Vesicles

The surface of the vesicles was further coated with 0.15% *w*/*v* chitosan solution. Chitosan was dissolved in 0.1% acetic acid and added dropwise to prepare IPI vesicles with continuous stirring. The samples were further incubated for 1 h with stirring [24]. Finally, the samples were sonicated with a probe sonicator for 1 min at 5 min time intervals in an ice bath for size reduction and stored in a glass vial for further characterization.

#### 2.2.3. Vesicle Characterization

Malvern computerized inspection system (Zetasizer, Malvern; United Kingdom) was used to determine size, PDI, and zeta potential. It works by the dynamic light scattering (DLS) method, which probes the hydrodynamic mobility of the particles. Polydispersity index (PDI) was measured for extent of size distribution measurement. The zeta potential was evaluated to check the surface charge of the vesicles and the standard value for the PDI and zeta potential is less than 0.7 and ±30 mV, respectively [25,26]. The samples were evaluated by diluting 0.1 mL dispersion in double distilled water (100-folds). The sample transferred to cuvette and their size, PDI, and zeta potential measured at room temperature. Transmission electron microscope study was performed by taking one drop of sample on carbon coated grid. The sample was stained with uranyl acetate and kept aside for few minutes. The stained sample air dried and visualized under high resolution microscope (JEOL, MA, USA). 

#### 2.2.4. Encapsulation Efficiency

The encapsulation efficiency of the prepared IPI vesicles was determined by the centrifugation method [27]. The difference between total IPI added and the concentration of IPI found in the vesicles was calculated. The formulations (2 mL) were transferred to centrifuge tube and centrifuged at 10,000 rpm for 30 min. The supernatant was collected and diluted to measure the absorbance. The absorbance of the sample was read at 296 nm and the entrapment efficiency was calculated using the formula [24].

Entrapment efficiency (EE%) = [(A − B)/A] × 100(1)

A = Initial amount of IPI added; B = Amount of IPI in supernatant solution.

#### 2.2.5. Drug Release

The release of IPI from the prepared IPI vesicles (F1, F2, F3, F4), IPI chitosan vesicle (F3C), was determined by using the dialysis method [20]. Each sample (~5 mg IPI) was filled in a dialysis bag (molecular weight 12 kDa) and tied from both ends. The bag was then placed in release media (500 mL), with continuous stirring (100 rpm) at a temperature of 37 ± 0.5 °C. Then, 2 mL samples were collected and the same volume of the fresh medium was added to maintain the study condition at all time points. The collected samples were evaluated to measure the amount of drug release at each time point by taking absorbance at 296 nm using a UV spectrophotometer (Shimadzu 1700, Kyoto, Japan).

(2) $$\mathrm{Drug}\;\mathrm{release}\;\left(\%\right)=\frac{\left(\mathrm{Released}\;\mathrm{concentration}\;x\;\mathrm{volume}\;\mathrm{of}\;\mathrm{media}\;x\;\mathrm{dilution}\right)}{\mathrm{Initial}\;\mathrm{concentration}\;}\;\times \;100$$

#### 2.2.6. Permeation Study

The prepared IPI vesicles (F1, F2, F3, F4), IPI chitosan vesicle (F3C), and pure IPI (~2 mg of IPI) were filled along the mucosal side of the goat intestinal. The goat intestine was collected from the local slaughterhouse and washed with ringer solution to remove the food residue. The intestines were stored at 4 °C till further use. The intestines were cut from both sides, the samples filled and tied. The temperature was fixed at 37 °C with the necessary conditions of aeration for the study. The sac was then immersed in Kreb’s solution (100 mL). The permeated sample (2 mL) was collected at a specified time interval (0, 1, 2, 3, 4, 6 h) and replaced with same amount of fresh Kreb’s solution [28]. The released content was filtered using a 0.2 µm membrane filter and the amount of drug permeation was determined using the HPLC method (Agilent 1260, Santa Clara, USA). The measurements were performed using the column Develosil C18, 4.6 × 150 mm, mobile phase composition of acetonitrile: phosphoric acid 0.2% (50:50), a flow rate of 1 mL/min and data estimated at 290 nm.

#### 2.2.7. Mucoadhesive Efficiency

The comparative adsorption study between IPI chitosan vesicles (F3C) and IPI vesicles (F3) was determined using mucin solution to evaluate the mucoadhesive property [29]. The mucin stock solution (1% *w*/*v*) was prepared and mixed with IPI chitosan vesicles (F3C) and IPI vesicles (F3) in a 1:1 ratio. The samples were kept aside for 1 h at 37 °C and then centrifuged for 60 min at 10,000 rpm. The supernatant was collected, diluted, and free mucin content was determined spectrophotometrically at 258 nm. The mucin concentration was calculated by using the below formula.

Mucoadhesive efficiency = [(C_o_ − C_F_)/C_o_] × 100 (3)

C_o_ = mucin initial concentration; C_F_ = free mucin.

#### 2.2.8. In Vivo Study

##### Animals

Experimental animals (Swiss albino mice and Wistar albino rats) were obtained from Bilwal Medchem and Research Pvt Ltd. Jaipur, Rajasthan, India. Animals of the same-sex were kept in separate cages in groups of three animals. The animals were maintained at the standard condition of temperature, lighting, and humidity, and were fed with a standard diet (Golden feed, New Delhi, India) and water ad libitum. The animal experimentation was approved by the Institutional Animal Ethics Committee (IAEC) of Bilwal Medchem and Research Pvt Ltd., Jaipur (Ethical number: BMRL/2020-14). For in vivo tail bleeding time, Swiss albino mice were divided into five groups, containing six animals in each group, used for platelet aggregation assay. Albino Wistar rats were divided into six groups, containing six animals (*n* = 6) in each group, to study the carrageenan-induced tail vein thrombosis. 

#### 2.2.9. Anti-Platelet and Anti-Thrombotic Study 

##### In Vivo Tail Bleeding Time

Swiss albino mice were divided into five groups for the study. Group I (control) received distilled water; group II administered aspirin (50 mg/kg); group III received pure IPI (20 mg/kg); group IV received IPI vesicles (F3, 20 mg/kg); group V received IPI chitosan vesicle (F3C) for seven days via oral route. After administration of the dose (1 h), the tail of the mice was removed 2 mm from the tip. Afterwards, the bleeding tip was dried over blotting paper every 30 s and the time was noted when the bleeding stopped [30,31].

##### In Vitro Anti-Thrombin Activity

The antithrombin activity was performed with five groups of each formulation. Group I served as negative control (no treatment); group II served as standard (aspirin 50 µL/mL); group III served as test group 1 (pure IPI 20 µL/mL); group IV served as test group 2 (IPI vesicles 20 µL/mL); group V served as test group 2 (IPI chitosan vesicles 20 µL/mL). The concentrate for each formulation was made by dissolving in a solvent (20 µL of pure IPI was dissolved in 1 mL of methanol). Then, 50 µL of each concentrate was added with 50 µL of Tris-HCl buffer, (pH 7.4) and 50 µL of thrombin in 96 well plates. This was followed by incubation at 37 °C for 5 min, followed by the addition of 50 µL of a chromogenic substrate, D-Phe-L-Pipecoyl-Arg p-nitroaniline, (prepared by dissolving 25 mg of the reagent in 20 mL of distilled water) was taken in each well. The absorbance is noted at 450 nm using a spectrophotometer. The anti-thrombin activity (%) was calculated using the below equation.

(4) $$\%\;\mathrm{Anti}-\mathrm{thrombin}\;\mathrm{activity}=(\frac{\mathrm{Absorbance}\;\mathrm{of}\;\mathrm{Negative}\;\mathrm{control}\;-\mathrm{Absorbance}\;\mathrm{of}\;\mathrm{test}\;\mathrm{sample}\;}{\mathrm{Absorbance}\;\mathrm{of}\;\mathrm{negative}\;\mathrm{control}})\;\times \;100$$

#### 2.2.10. Platelet Aggregation Assay

The study was performed on Wistar albino rats and divided into six groups. Group I served as control; group II served as negative control; group III served as standard (aspirin 50 mg/kg); group IV received (pure IPI 20 mg/kg); group V treated with IPI vesicle (F3), 20 mg/kg); group VI treated with IPI chitosan vesicles (F3C, 20 mg/kg). Each experimental group received their respective treatment for five days via oral route. Then, 2 h following the last dose, blood samples from each rat was collected and serum was isolated. The platelets were prepared as per the reported procedure [32] and added to each tube containing serum in concentration 250 µL/mL. The tubes were incubated for 120 s at 37 °C, and then 0.25 U/mL of thrombin was added. After 20 min, the absorbance of the samples was noted at 600 nm. The percentage inhibition of platelet aggregation was calculated using the formula [33]:

(5) $$\mathrm{Inhibition}\;\mathrm{of}\;\mathrm{platelet}\;\mathrm{aggregation}\;\left(\%\right)=\left(\frac{\mathrm{Absorbance}\;\mathrm{of}\;\mathrm{test}\;-\mathrm{Absorbance}\;\mathrm{of}\;\mathrm{control}\;}{\mathrm{Absorbance}\;\mathrm{of}\;\mathrm{test}}\right)\times 100$$

Percent anti-platelet activity was calculated as:

(6) $$\mathrm{Percent}\;\mathrm{antiplatelet}\;\mathrm{activity}=\left(\frac{\mathrm{Percent}\;\mathrm{inhibition}\;\mathrm{of}\;\mathrm{sample}}{\mathrm{Percent}\;\mathrm{inhibition}\;\mathrm{of}\;\mathrm{Standard}}\right)\times 100$$

#### 2.2.11. Carrageenan-Induced Tail Vein Thrombosis

Wistar albino rats were divided into five groups. Group I served as negative control; Group II served as standard and received Aspirin 50 mg/kg, p.o; group III served as test group 1 (Pure IPI 20 mg/kg); group IV served as test group 2 (IPI vesicles, F3, 20 mg/kg); group V served as test group 3 (IPI chitosan vesicles, F3C, 20 mg/kg). The treatment groups were administered with their respective treatment for 3 days via an oral route. After 1 h of the administration of last dose, the animals were anaesthetized using ketamine hydrochloride 100 mg/kg, i.p. Afterwards, Carageenan 20 mg/kg, i.v was administered to each rat of the experimental group. Subsequently, the tail of the rats was dipped into ice-cold water for 2 min and ligated from the tip of the tail for 20 min. Afterward, the ligature was removed and the animals were observed for few hours for possible swelling, redness, and a characteristic thrombus formation. Further, the animals were observed for 24 and 48 h and thrombus length was measured for each group [34,35].

#### 2.2.12. Statistical analysis

Graph pad prism 9.2.0 was used for analysis of data. Data are expressed as Mean ± SEM and analysed using a one-way ANOVA followed by Dunnett’s test of multiple comparison. **** *p* ≤ 0.0001, *** *p* ≤ 0.001, ** *p* < 0.01, and * *p* ≤ 0.05 represent statistical significance.

## 3. Results and Discussion

IPI nanovesicles were prepared by solvent evaporation–hydration method. The vesicles were prepared by using cholesterol, phospholipid, and different types of spans (non-ionic surfactants) to evaluate the size, encapsulation efficiency, drug release, and permeation flux to select the optimized IPI vesicles. On the basis of minimum vesicle size, zeta potential, high encapsulation efficiency, and drug release (Table 2), the formulation (F3) is selected as the optimized IPI vesicles used for further coating with chitosan. The chitosan coated vesicle (F3C) was further evaluated for muco-adhesiveness, drug release, drug permeation, and in vivo animal studies (antithrombotic and anti-platelet activity). 

### 3.1. Vesicle Characterization

**Figure 1:**
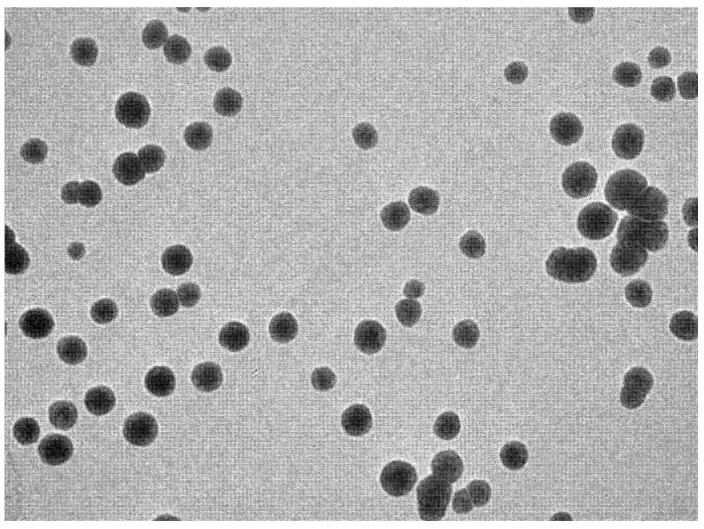
Surface morphology of IPI chitosan vesicles using a transmission electron microscope.

The prepared formulations (IPI vesicles and IPI chitosan vesicles) were evaluated for size, PDI, and zeta potential. The prepared IPI vesicles and IPI chitosan vesicles (F3C) showed a size between 270 ± 6.2 nm (F1) and 379 ± 4.5 nm (F3C), as shown in Table 2. The size of particles/vesicles must be less than 500 nm to transport the drugs through the endocytosis pathway [36]. The prepared formulation (F1–F3C) showed a size below 500 nm. This size can also help to enhance the drug absorption due to the availability of a greater surface area. The formulation (F4) prepared with span 80 (352 ± 5.9 nm) has shown the large size vesicles and the formulation prepared with span 20 showed the smallest size vesicles (270 ± 6.2 nm). The increase in the hydrophilic lipophilic balance (HLB) value of surfactant leads to a larger vesicle size [37]. The selected formulation (F3) was further coated with chitosan (F3C) and showed a significant increase in vesicle size with an average diameter of 379 ± 4.5 nm. There was a significant (*p* < 0.05) variation in the size due to the use of different types of non-ionic surfactants. This value showed that the chitosan coating increased the vesicle size by forming a thin layer over the IPI vesicles (F3). The vesicle size distribution (PDI) data of the prepared samples (F1–F3C) showed a narrow size distribution (0.13 to 0.36). The low PDI value confirms the homogeneity of prepared vesicles, the PDI value less than 0.7, gives the uniform size distribution [25]. The zeta potential of IPI vesicles was found between -18.7 and + 26.4 mV, and the value ± 30 mV, considered as the standard range, gives a stable formulation [26]. The negative charge on the vesicles is due to the negative charge of the lipid [38]. High negative zeta potential of the prepared samples indicates superior stability. The sample coated with chitosan gives the positive zeta potential (+23.5 mV) value. The zeta potential significantly changes from negative to positive due to the cationic charge of chitosan. The presence of a positively charged amine group of chitosan is responsible for positive zeta potential [39]. The positive charge of chitosan easily binds with negatively charged intestinal mucin and will help to increase drug properties [40]. The formulation surface also evaluated by the TEM and the image showed spherical shape vesicles (Figure 1). The vesicles were found to be non-aggregated and spherical in shape. The non-aggregated particles support the low PDI value and optimum zeta potential. It confirms the stability of particles.

### 3.2. Encapsulation Efficiency

The prepared IPI vesicles (F1–F4) and IPI chitosan vesicles (F3C) formulations showed the encapsulation efficiency from 68.4 ± 4.1% (F1) to 86.2 ± 5.3% (F3). The surfactant nature and surfactant to cholesterol ratio are the important parameters to affect the EE%. The result of encapsulation efficiency is depicted in Table 2. The difference in the encapsulation efficiency is found to be significant (*p* < 0.05) by using a different non-ionic surfactant. The maximum EE was found from the formulation (F3) prepared from the span 60 due to the longer alkyl chain length and increased lipophilicity of the enclosing environment [41]. The formulation (F1) prepared with span 20 as surfactant depicted the lowest EE. The chitosan-coated IPI vesicles (F3C) showed slightly lesser encapsulation efficiency (77.3 ± 4.1%) than IPI vesicles (F3). The reason for the lesser encapsulation is the drug does not completely come out from the vesicles and these results support the slow drug release from chitosan-coated formulations. Chitosan formed a surface coating over the lipid bilayer of liposomes and prevents the leakage of the drug [42]. 

### 3.3. Drug Release

**Figure 2:**
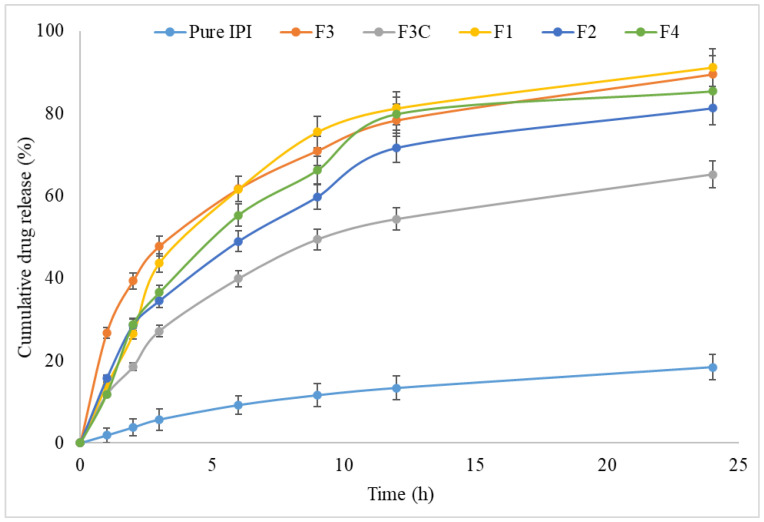
In vitro drug release profile of IPI vesicles (F1, F2, F3, F4) and IPI chitosan vesicle (F3C). Study performed in triplicate and data shown as mean ± SD.

The formulation composition mainly affects the drug release pattern. The amount of drug released from the IPI vesicles (F1–F4) and IPI chitosan vesicles (F3C) showed marked differences in release pattern (Figure 2). The maximum drug release was shown by the formulation F1 prepared with span 20 as a surfactant (91.2 ± 2.56%). The maximum drug release was achieved due to the higher hydrophilic nature of span 20. After completion of the study, the release was found in the pattern of IPI vesicles (81.3 ± 3.3 to 91.2 ± 2.6%) > IPI chitosan vesicle (64.2 ± 3.2%). The release pattern showed biphasic release behaviour from IPI chitosan vesicles and IPI vesicles. The formulations F1 to F4 showed fast release between 26.46 ± 2.7% and 39.3 ± 2.6% in the initial 2 h, and later prolonged release was achieved till the completion of the study. A significant difference in the IPI release was found in the initial 2 h between the formulation (F3) and formulations (F1, F2, F4). The surfactant in the IPI vesicles helps to solubilize the drug in release media. The fast release occurred due to the availability of drug on the vesicles surface and later the slower release was found. The slower release is due to the encapsulated drug in the lipid bilayer and released by the diffusion and erosion or swelling of the carrier [43]. The chitosan-coated vesicles showed lower drug release than the IPI vesicles. The formulation (F3C) showed lesser IPI release (18.23 ± 1.8%) in the initial 2 h. The release was found to be significantly lesser than the uncoated vesicles. The maximum drug release was found to be 64.2 ± 3.1% in 24 h and was significantly lower than other formulations. The reason for the slow drug release is that the drug needs to cross the double layer (lipid and then chitosan layer) in order to disperse out in the release media. The negative surface of the vesicles coated with chitosan via electrostatic interaction leads to a reduction in the drug release [42]. The release study results fitted into different kinetic release models and the correlation coefficient value was found higher for the Higuchi model (R^2^ = 0.9916). The drug release followed the diffusion mechanism to release the content. This model also helps to interpret the mechanism of water-soluble and insoluble drugs from unilamellar and multilamellar niosomes. In this study, the Korsmeyer–Peppas equation was utilized to interpret the release kinetics. The permeability exponent value was found to be 0.51, which was found in the reference range of 0.45–0.89 [44]. 

### 3.4. Mucoadhesive Efficiency

**Figure 3:**
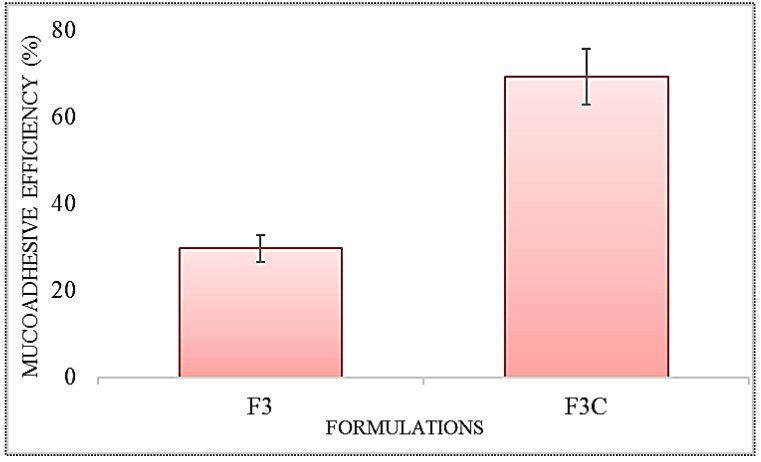
Mucoadhesive efficiency study results of IPI vesicles (F3) and chitosan-coated IPI vesicles (F3C). The results shown in triplicate and data shown as mean ± SD.

The mucoadhesive efficiency of the IPI vesicles (F3) and chitosan-coated IPI vesicles (F3C) was assessed based on mucin adsorption characteristics. The result of the study revealed a significant enhancement in muco-adhesion from the IPI chitosan vesicle (F3C), as shown in Figure 3. The chitosan vesicles (F3C) showed the muco-adhesive efficiency of 68.76 ± 3.45%, whereas the formulation F3 showed significantly lesser (*p* < 0.05) muco-adhesive efficiency 29.53 ± 2.11%. The enhancement was found to be 2.3-fold higher than IPI vesicles (F3). The enhanced muco-adhesion property is shown by the IPI chitosan vesicles (F3C) due to the surface coating of IPI vesicles with chitosan. The greater binding of chitosan with mucin is due to electrostatic interaction between them. The cationic charged chitosan and negatively charged mucin interact with each other, as well as hydrogen bonding and hydrophobic interaction lead to greater binding. It indicates that chitosan IPI vesicles have shown better muco-adhesive properties, longer residence time in the GIT region and also helps to obtain better therapeutic efficacy [45].

### 3.5. Permeation Study

**Figure 4:**
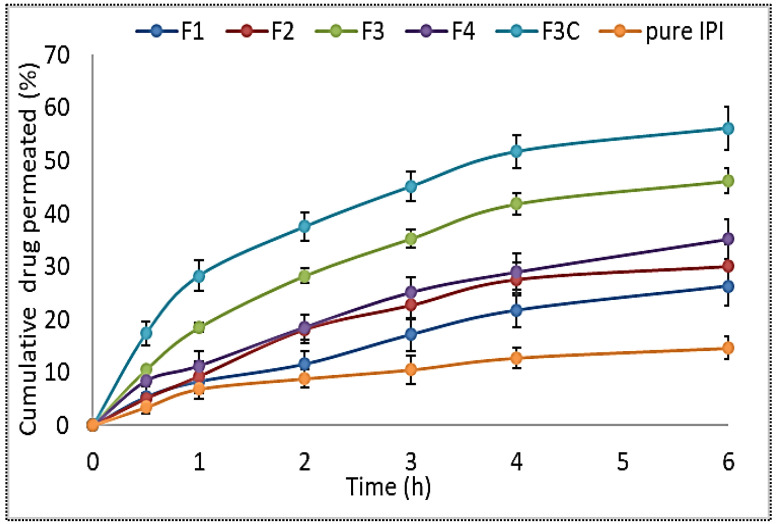
Cumulative drug permeation of IPI vesicles (F1, F2, F3, F4), IPI chitosan vesicle (F3C), and pure IPI. Study performed in triplicate and data shown as mean ± SD.

The results of the permeation study were performed using goat intestine and the results depicted in Figure 4. The cumulative amount of drug permeated from IPI vesicles (F1–F4) and IPI chitosan vesicle (F3C) revealed a significant (*p* < 0.05) difference than the pure IPI. The pure IPI showed lower permeation flux of 0.43 ± 0.11 µg/cm^2^/h. The prepared formulations IPI vesicles (0.74 ± 0.21 µg/cm^2^/h to 0.98 ± 0.31 µg/cm^2^/h) and IPI chitosan vesicle (F3C, 1.11 ± 0.31 µg/cm^2^/h) showed significantly greater drug permeation. The pure IPI showed significantly lower permeation (*p* < 0.05) than IPI vesicles (F3) and IPI chitosan vesicles (F3C). The result between IPI vesicles (F3) and IPI chitosan vesicles (F3C) also revealed a significant difference in the permeation profile. IPI chitosan vesicles (F3C) showed the enhancement ratio of 2.6-fold to pure IPI, whereas the formulation F3 showed 2.3-fold enhancement in permeation. The enhancement in permeation from the formulation F3 is due to the presence of surfactant in the vesicles which helps to solubilize the poorly soluble drug. The presence of cholesterol and lipid in the vesicles also help to reduce the barrier property at the site of absorption [46]. In the case of chitosan-coated vesicles (F3C), the chitosan helps to enhance the mucoadhesive property and also leads to the disruption of the tight junction of the mucosa. The presence of a positive charge of the chitosan amino group interacts with the negative charge of sialic acid of the intestinal membrane. Thus, it gives greater permeation across the mucosal surface [45].

### 3.6. In Vivo Tail Bleeding Time

**Figure 5:**
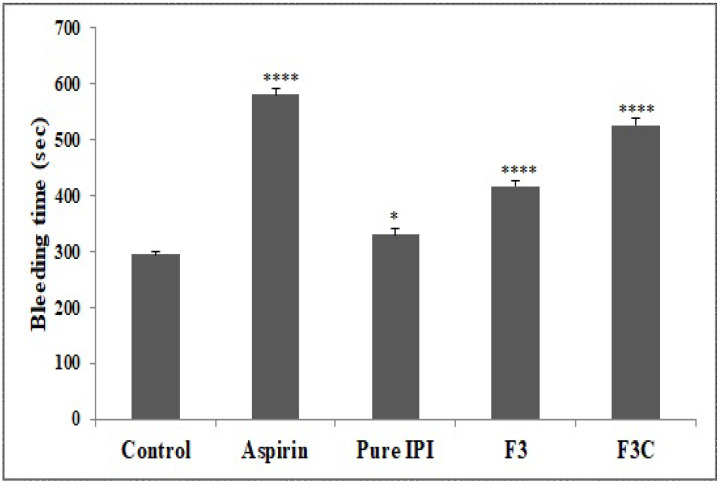
Effect of various treatments on tail bleeding time in experimental animals. Results are expressed as Mean ± SEM, (*n* = 6), analysed by one-way ANOVA followed by Dunnett’s test of multiple comparison where ns = non-significant, **** *p* < 0.0001, * *p* < 0.05 where various treatment groups were compared with control group.

In the present study, the bleeding time was assessed using a template model and the results are shown in Figure 5. IPI chitosan vesicles (F3C) and IPI vesicles (F3) significantly (*p* < 0.0001) prolonged the bleeding time from 295 ± 5.63 s (control) to 416.7 ± 9.5 s (F3) and 526.5 ± 11.9 s (F3C). The pure IPI showed the slightly increased bleeding time 330.2 ± 10.49 s. The enhancement in the bleeding time was found to be slightly significant (*p* < 0.05). The template bleeding time shed light on platelet participation in primary haemostasis [3,47], and evaluates the effects of administered drug to maintain the haemostasis [48]. The standard drug Aspirin, IPI vesicle (F3), and IPI chitosan vesicle (F3C) are capable of prolonging the bleeding time. However, the impairment of haemostasis is found to be lesser with Pure IPI. The lower effect found may be due to the poor solubility and dissolution of pure IPI. The prolonged bleeding time is correlated with low platelet counts and the involvement of platelet as an integral component of haemostatic response [49]. Here, the prolonged bleeding time suggests the inhibition of platelet aggregation [47].

### 3.7. Anti-Thrombin Activity

**Figure 6:**
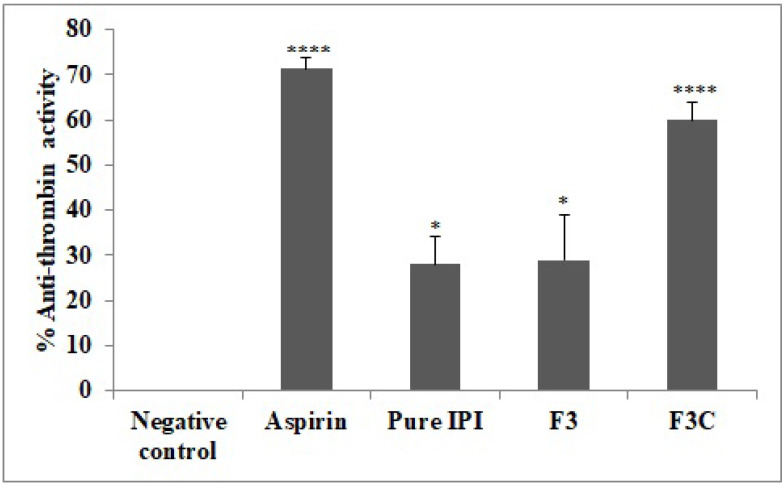
Effect of various treatments on percent anti-thrombin activity. Results are expressed as Mean ± SEM (*n* = 6), analysed by one-way ANOVA followed by Dunnett’s test of multiple comparison where ns = non-significant, **** *p* < 0.0001, * *p* < 0.05 where various treatment groups were compared with negative control.

The comparative antithrombin activity was performed on different groups and the results are depicted in Figure 6. Aspirin and IPI chitosan vesicles (F3C) displayed highly significant (*p* < 0.0001) anti-thrombin activity, i.e., 71.2 ± 3% and 60 ± 4% as compared to a negative control group, whereas pure IPI and IPI vesicles (F3) showed the lower activity of 28 ± 6% and 29 ± 10%, but were significantly (*p* < 0.05) higher in comparison to the negative control group. Enzyme thrombin plays a pivotal role in the process of blood clotting and, hence, its inhibition is very crucial in counteracting various blood clotting and platelet disorders. The main reason to target thrombin for the inhibition of coagulation is the clotting of fibrinogen and rendering the formation of thrombus. Therefore, the fact that thrombin inhibitors can effectively attenuate thrombus formation does not affect other coagulant enzymes. The thrombin enhances smooth muscle proliferation and mobilization by acting as a pro-inflammatory factor which ultimately causes more platelet activation. However, anti-thrombotic agents encourage prostaglandins secretion from vasculo-endothelial cells, hence, attenuating the activation of platelets [48]. 

### 3.8. Platelet Aggregation Assay 

**Figure 7:**
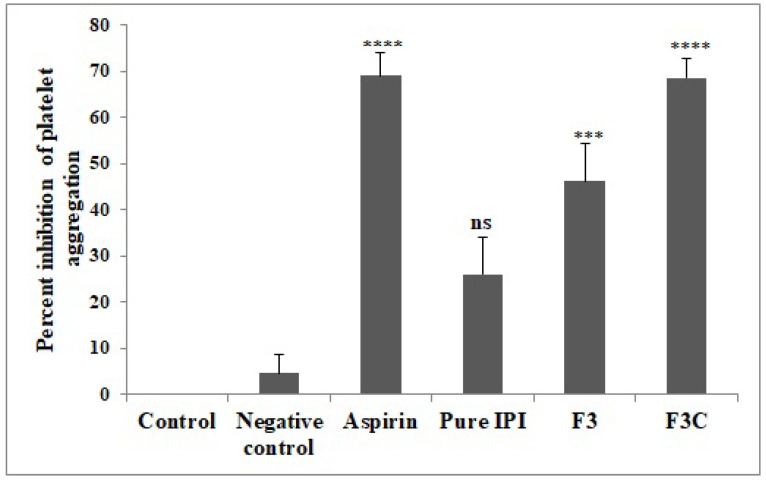
Effect of various treatments on percent inhibition of platelet aggregation. Results are expressed as Mean ± SEM (*n* = 3), analysed by one-way ANOVA followed by Dunnett’s test of multiple comparison where ns = non-significant, **** *p* < 0.0001, *** *p* < 0.001, *p* < 0.05 where various treatment groups were compared with negative control group.

Platelet aggregation assay is commonly used to evaluate the anti-platelet and anti-thrombotic activity [49] and the results are shown in Figure 7. The results clearly showed that the inhibition of platelet aggregation and antiplatelet activity of IPI chitosan vesicles (F3C) was higher than pure IPI and IPI vesicles (F3). There is a significant (*p* < 0.0001, *p* < 0.001) inhibition was observed with IPI chitosan vesicles and IPI vesicles, as compared to a negative control group. The antiplatelet activity was found to be 68.6 ± 4% (F3C) and 46.2 ± 8% (F3), respectively. The antiplatelet activity was almost equal to that of the standard drug Aspirin. It showed anti-platelet activity of 69 ± 5.4%. The pure IPI (26 ± 8.1%) showed non-significant inhibition of platelet aggregation as compared to the negative control. Thrombin-induced platelet aggregation is based on hydroxylation of the protease-activated receptor on the platelet resulting in its activation and exposure of a new N-terminal sequence. Furthermore, a tethered ligand leads to the initiation of a sequence of the event resulting in thrombus formation. Thrombin also contributes to platelet aggregation by activation of the platelet pathway through GP1b [50,51,52]. 

### 3.9. Carageenan Induced Tail Vein Thrombosis

The results showed greater activity of IPI chitosan vesicles (F3C) than pure IPI and IPI vesicles (F3), as shown in Table 3. The thrombus length of rats in the negative control group was found to be 12.51 ± 0.4 cm and 12.7 ± 0.4 cm at 24 h and 48 h, respectively. The IPI chitosan vesicles (F3C) treated group depicted significantly (*p* < 0.0001) reduced thrombus length to 9.4 ± 0.2 cm and 8.95 ± 0.2 cm at 24 h and 48 h, respectively. Similarly, Aspirin treatment showed reduced thrombus length of 10.6 ± 0.4 cm (*p* < 0.001) and 9.73 ± 0.3 cm (*p* < 0.0001) compared to negative control at 24 h and 48 h, respectively. The data clearly showed that IPI chitosan vesicle (F3C) was found to be more effective than Aspirin in the reduction in thrombus length. IPI vesicles reduced the thrombus length to 10.98 ± 0.3 cm at 48 h (*p* < 0.01) and does not reduce the thrombus length at 24 h, compared to a negative control group. The pure IPI was found to be less effective in reducing thrombus length both at 24 h and 48 h compared to a negative control. A similar type of findings of Allyl isothiocyanate (AITC) was reported in rats using platelet aggregation assay and in vivo tail-bleeding time. The result revealed that AITC showed significant inhibition of platelet aggregation at a dose of 3 mg/kg. The inhibition of platelet aggregation in response to thrombin was found between 15% and 20%. On the other hand, the inhibition of platelet aggregation by IPI chitosan vesicle and IPI vesicle was found to be 68.6% and 46.2%, respectively, which is much higher than that of AITC.

## 4. Conclusions

IPI chitosan vesicle was prepared using the solvent evaporation method to evaluate *in vitro* and *in vivo* activities. The prepared IPI vesicles showed nano-vesicle size, low PDI and high encapsulation efficiency. Among the prepared IPI vesicles, the formulation (F3) was selected as an optimized formulation with vesicle size (298 ± 5.1 nm), PDI (0.33), zeta potential (−18.7 mV), and encapsulation efficiency of 86.2 ± 5.3% was further coated with chitosan. The IPI chitosan vesicles (F3C) depicted enhanced permeation and mucoadhesive activity. The IPI chitosan vesicle (F3C) revealed significantly enhanced flux (0.98 ± 0.14 µg/cm^2^/h) and mucoadhesive property. The comparative release profile showed enhanced and biphasic drug release. The formulation (F3C) also evaluated for *in vivo* antiplatelet and anti-thrombotic activity and results showed significantly reduced thrombus length, platelet aggregation, and high anti-thrombin activity than the other treatment groups. The results were found to be closer to the standard aspirin and further preclinical and clinical study needs to validated to establish IPI vesicles as delivery systems. 

## Figures and Tables

**Table 1:** Formulation composition of IPI vesicles and IPI chitosan vesicles.

Code	Cholesterol (%)	Span 20 (%)	Span 40 (%)	Span 60 (%)	Span 80 (%)	Phospholipid (%)	Chitosan (*w*/*v*)
F1	10	10	0	0	0	80	0
F2	10	0	10	0	0	80	0
F3	10	0	0	10	0	80	0
F4	10	0	0	0	10	80	0
F3C	10	0	0	10	0	80	0.15

**Table 2:** Physicochemical evaluation results of IPI vesicles and IPI chitosan vesicles. Each study performed in triplicate and the data shown as mean ± SD, (*n* = 3).

Code	Surfactant	Size(nm)	PDI	Zeta Potential (mV)	Encapsulation Efficiency (%)	Drug Release (%)
F1	span 20	270 ± 6.2	0.13 ± 0.05	−21.5 ± 1.5	68.4 ± 4.1	91.2 ± 4.9
F2	span 40	311 ± 5.5	0.24 ± 0.04	−26.4 ± 1.7	70.6 ± 4.6	86.3 ± 3.2
F3	span 60	298 ± 5.1	0.33 ± 0.07	−18.7 ± 1.8	86.2 ± 5.3	89.5 ± 3.5
F4	span 80	352 ± 5.9	0.36 ± 0.06	−24.5 ± 2.4	74.6 ± 3.5	81.3 ± 3.3
F3C	span 60	379 ± 4.5	0.32 ± 0.08	+23.5 ± 2.8	77.3 ± 4.1	63.2 ± 3.9

**Table 3:** Effect of various treatments on thrombus length in Carageenan induced tail vein thrombosis. Data are shown as Mean ± SEM, (*n* = 6). ^ns^ = non-significant, **** *p* < 0.0001, *** *p* < 0.001, ** *p* < 0.01, where various treatment groups were compared with negative control group.

S. No	Treatment and Dose (BW)	Parameters		
		Tail Length (cm)	Thrombus Length (cm) 24 h	Thrombus Length (cm) 48 h
1	Negative control	13.83 ± 0.3	12.51 ± 0.4	12.7 ± 0.4
2	Aspirin (50 mg/kg)	14.05 ± 0.3 ^ns^	10.6 ± 0.4 ***	9.73 ± 0.3 ****
3	Pure IPI (20 mg/kg)	13.95 ± 0.2 ^ns^	12.01 ± 0.3 ^ns^	11.89 ± 0.2 ^ns^
4	F3 (20 mg/kg)	13.96 ± 0.3 ^ns^	11.76 ± 0.2 ^ns^	10.98 ± 0.3 **
5	F3C (20 mg/kg)	13.83 ± 0.3 ^ns^	9.4 ± 0.2 ****	8.95 ± 0.2 ****

## Data Availability

Not applicable.
